# Real-Time Visualization of a Focused Ultrasound Beam Using Ultrasonic Backscatter

**DOI:** 10.1109/TUFFC.2020.3035784

**Published:** 2021-03-26

**Authors:** Miles Thies, Michael L. Oelze

**Affiliations:** Department of Electrical and Computer Engineering, Beckman Institute for Advanced Science and Technology, University of Illinois at Urbana–Champaign, Urbana, IL 61820 USA

**Keywords:** Beam visualization, focused ultrasound (FUS), real-time, therapy monitoring

## Abstract

Focused ultrasound (FUS) therapies induce therapeutic effects in localized tissues using either temperature elevations or mechanical stresses caused by an ultrasound wave. During an FUS therapy, it is crucial to continuously monitor the position of the FUS beam in order to correct for tissue motion and keep the focus within the target region. Toward the goal of achieving real-time monitoring for FUS therapies, we have developed a method for the real-time visualization of an FUS beam using ultrasonic backscatter. The intensity field of an FUS beam was reconstructed using backscatter from an FUS pulse received by an imaging array and then overlaid onto a B-mode image captured using the same imaging array. The FUS beam visualization allows one to monitor the position and extent of the FUS beam in the context of the surrounding medium. Variations in the scattering properties of the medium were corrected in the FUS beam reconstruction by normalizing based on the echogenicity of the coaligned B-mode image. On average, normalizing by echogenicity reduced the mean square error between FUS beam reconstructions in nonhomogeneous regions of a phantom and baseline homogeneous regions by 21.61. FUS beam visualizations were achieved, using a single diagnostic imaging array as both an FUS source and an imaging probe, in a tissue-mimicking phantom and a rat tumor *in vivo* with a frame rate of 25–30 frames/s.

## I. Introduction

FOCUSED ultrasound (FUS) is a therapeutic modality with a broad range of clinical applications. Notable examples of FUS therapy include the treatment of uterine fibroids [1], bone metastases [2], essential tremor [3], and prostate cancer [4]. Furthermore, there has been a multitude of clinical and preclinical studies exploring applications of FUS to the treatment of other diseases [5–9].

FUS therapies operate by noninvasively inducing biological effects in localized regions of tissue using a tightly focused ultrasound beam. Therapeutic effects are achieved through thermal or mechanical mechanisms. Thermal-based FUS therapies use ultrasonic energy to produce temperature elevations in well-defined regions, typically for the surgical ablation of tissue. Mechanical-based FUS treatments induce therapeutic effects through mechanical stresses caused by the ultrasound wave. Some emerging applications of mechanical-based FUS are transient opening of the blood–brain barrier [10], targeted drug delivery [11], histotripsy [12], and sensitization of tumors to radiation therapy [13].

For both categories of therapy, it is crucial to continuously monitor the FUS beam during treatment to ensure that therapeutic effects are constrained to target regions, while healthy tissues are spared. There have been sustained research efforts devoted toward developing ultrasound techniques to monitor thermal-based FUS therapies, such as ultrasound thermometry [14], [15] and using hyperechogenicity in B-mode images [16–18]. However, these methods have not seen widespread use due to the superior imaging capabilities of magnetic resonance imaging (MRI), which is the clinical standard in FUS therapy monitoring. MRI can provide near real-time quantitative mapping of temperature changes in tissue [19]. MRI guidance is invaluable for thermal-based FUS therapies because it can be used for localization of the FUS beam, monitoring of thermal bioeffects, and closed-loop feedback control of energy deposition [20]. Boiling histotripsy, a mechanical-based FUS therapy can also use MRI thermometry for therapy guidance [21]. Although MRI-guided technologies have facilitated the clinical acceptance of FUS therapies, MRI is expensive and nonportable, and FUS systems must be designed for compatibility with MRI [22].

Many mechanical-based FUS therapies are ill-suited for MRI guidance because little to no temperature elevations are produced during treatment. Thus, there is a need for mechanical-based FUS monitoring techniques that do not rely on thermal mechanisms. Conventional B-mode imaging can be used for real-time monitoring of some mechanical-based therapies, such as histotripsy, because the formation of bubbles at the focus produces a hyperechoic region during treatment [23], [24]. Passive cavitation imaging (PCI) is another ultrasound-based technique that has generated interest in recent years [25–29]. PCI uses passive ultrasound arrays to detect emissions from cavitating microbubbles and can create quantitative images of acoustic cavitation activity. Active cavitation imaging can also be used to visualize microbubble populations [30–32]. Real-time monitoring of an *in vivo* mechanical-based FUS therapy was recently achieved using PCI [33]. However, PCI suffers from poor axial resolution due to the passive beamforming methods used. Traditional delay-and-sum (DAS) beamforming can be used to improve axial resolution, but this is restricted to therapies where short therapy pulses are used [30]. Furthermore, PCI is based on the detection of nonlinear emissions, and it typically requires a secondary ultrasound probe with a bandwidth that contains the harmonics of the therapy pulse although a mechanical-based FUS therapy was recently monitored using PCI with a single diagnostic imaging array [34].

While many ultrasound-based therapy monitoring techniques have been explored, minimal work has been devoted toward visualizing the actual FUS beam *in situ.* In the past work, our group presented a novel method for the *in situ* visualization of the intensity field of an FUS beam [35]. A single element FUS source and a diagnostic imaging array were used in a bistatic configuration (i.e., 90° angle between transducers). The imaging array had an interleaved pulse sequence, alternating between the passive recording of ultrasonic backscatter from the FUS beam and active B-mode imaging. The recorded backscatter was processed to reconstruct the intensity field of the focused beam, which was then overlaid onto a B-mode image to visualize the position of the beam in the context of the surrounding medium. Because therapeutic effects are induced where the FUS beam intersects with tissue, knowledge of the position of the focused beam can be used for qualitative monitoring of an FUS therapy.

The *in situ* beam visualizations in [35] were achieved using off-line processing (i.e., not real time), and the technique was not thoroughly tested in nonhomogeneous media. Toward the goal of developing a real-time FUS therapy monitoring technique, we have built on the method proposed in [35] and achieved real-time FUS beam visualizations. The novelty of this work is that a single diagnostic imaging array was used as both an FUS source and an imaging probe for real-time *in situ* visualization of the FUS beam using ultrasonic backscatter. To achieve suitable beam visualizations in nonhomogeneous media, we have developed a normalization method that uses a coaligned B-mode image to correct the beam visualizations for differences in the scattering properties of the medium. A consequence of normalizing by echogenicity was an amplification of sidelobes, which arose due to the image reconstruction algorithm and not the FUS beam itself. To mitigate the effects of sidelobes, the FUS beam visualizations were beamformed using DAS beamforming with coherence factor (CF) weighting, which is an adaptive beamforming technique for the reduction of sidelobes [36], [37]. Real-time beam visualizations, while carrying out a simulated FUS therapy, were demonstrated in nonhomogeneous regions of a tissue-mimicking phantom and in a rat tumor *in vivo.* Real-time beam visualizations using the proposed method were first demonstrated in [38]. In this study, the beam visualization technique is described in more detail, and the effects of normalizing by echogenicity are thoroughly addressed and quantified.

## II. Methods

### A. Overview of Excitation Sequence

**Fig. 1.**
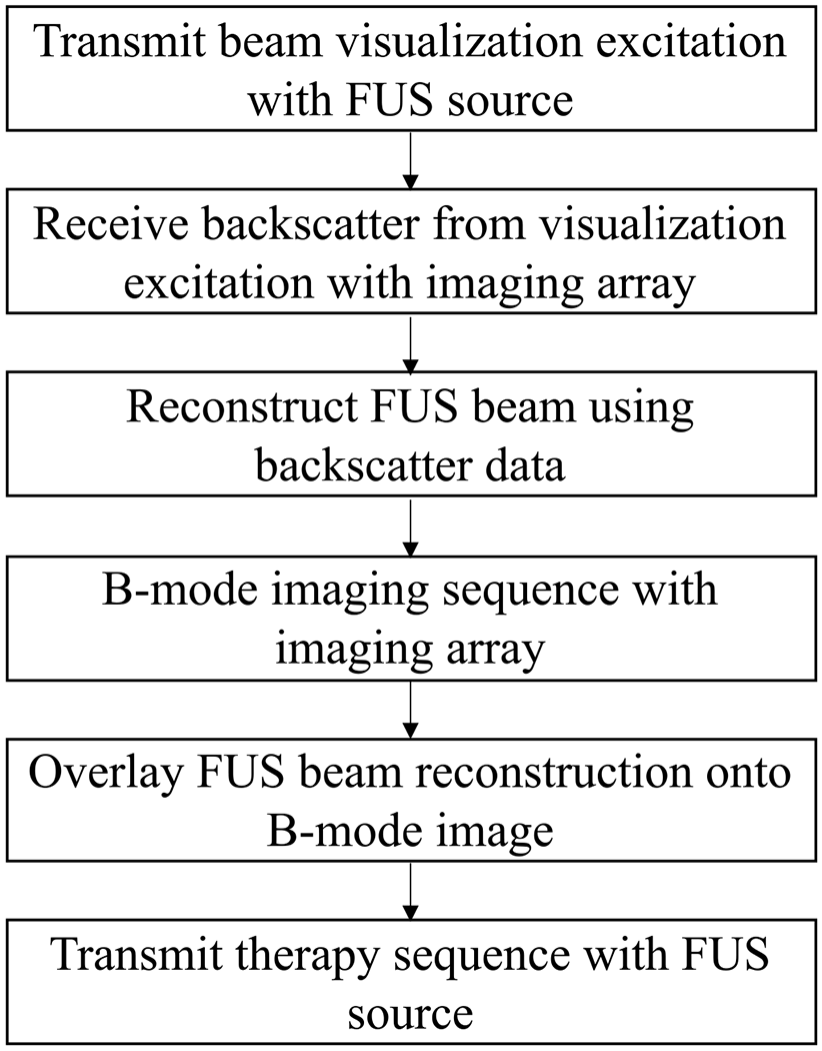
Flow diagram of beam visualization method for monitoring of an FUS therapy.

The flow diagram of the excitation sequence for the capture of an FUS beam visualization image is shown in Fig. 1. The excitation sequence requires an FUS source and an imaging array. In this work, a diagnostic imaging array was used for imaging and as the FUS source, but the same method could be used with two separate coaligned transducers. It is assumed that the FUS therapy does not have a 100% duty cycle. During the off-cycle of the therapy, the FUS source transmits a short focused pulse used for beam visualization, which is focused on the same location as the FUS therapy beam. The visualization pulse has a lower mechanical index than the therapy pulse to minimize any therapeutic effects. Backscatter from the visualization pulse is received by the imaging array and processed to create a visualization of the FUS beam. The imaging array then acquires a B-mode image of the area being treated, which is coregistered with the FUS beam reconstruction. Finally, the FUS beam visualization is superimposed onto the B-mode image to allow real-time monitoring of the beam’s position relative to the surrounding medium.

### B. FUS Beam Visualization

Using the model outlined in [39] and [40], the received signal $\upsilon \left(t,\vec{r}\right)$ at the imaging array after the FUS source transmits a focused visualization pulse can be expressed as

(1) $$\upsilon \left(t,\vec{r}\right)=\upsilon_{i}\left(t\right)*e_{T}\left(t\right)*h_{T}\left(t,\vec{r}\right)*s\left(t,\vec{r}\right)*h_{R}\left(t,\vec{r}\right)*e_{R}\left(t\right)$$

where * denotes convolution with respect to time, *v_i_* (*t*) is the excitation pulse, *e_T_* (*t*) and *e_R_*(*t*) are the electromechanical impulse responses of the transducers during transmission and reception, $h_{T}\left(t,\vec{r}\right)$ and $h_{R}\left(t,\vec{r}\right)$ are the spatial impulse responses of the transducers during transmission and reception, and $s\left(t,\vec{r}\right)$ is a scattering function that accounts for inhomogeneities in the medium. This model does not account for dispersive attenuation. The goal of the proposed method is to find the actual transmitted FUS field $p\left(t,\vec{r}\right)$, which is

(2) $$p\left(t,\vec{r}\right)=\upsilon_{i}\left(t\right)*e_{T}\left(t\right)*h_{T}\left(t,\vec{r}\right).$$

To derive a suitable estimate of $p\left(t,\vec{r}\right)$, the spatial dependence of $s\left(t,\vec{r}\right)$ and $h_{R}\left(t,\vec{r}\right)$ must be removed from $u\left(t,\vec{r}\right)$. By using dynamic receive focusing and a dynamic receive aperture during beamforming, $h_{R}\left(t,\vec{r}\right)$ can be made nearly spatially invariant, allowing us to express it as *h_R_(t)*. The scattering function $s\left(t,\vec{r}\right)$ can be accounted for by normalizing the beamformed data using the coaligned B-mode image, which visualizes $s\left(t,\vec{r}\right)$. Thus, we arrive at an estimate of the FUS pressure field $p\left(t,\vec{r}\right)$ that is smoothed due to a convolution of *e_R_(t)* with *h_R_(t)*. The intensity field of the FUS beam can then be calculated using the estimated pressure field $p\left(t,\vec{r}\right)$.

To reduce the effects of sidelobes on the beam visualization, backscattered echoes received by the imaging array from the visualization pulse were beamformed using DAS-CF beamforming. The real RF data were sampled at approximately four samples per wavelength. Linear interpolation was used for subsample delays to reduce quantization errors. A dynamic receive subaperture with a fixed f-number of 1 was centered around an element x_c_, and time delays *τ*(*x, z*) were calculated for each point (*x, z)* in the subaperture as follows:

(3) $$\tau \left(x,z\right)=\tau_{F}\left(x_{c}\right)+\frac{z+\sqrt{z^{2}+{\left(x-x_{c}\right)}^{2}}}{c}$$

where the *z*-axis is imaging depth, the *x*-axis is along with the array, *c* is the speed of sound in the medium, and *τ_F_* (*x_c_*) is the time delay applied on transmit to focus the center element *x_c_*.

After applying time delays, the receive subaperture RF data *s*(*x, z*) were apodized with a Hanning window and then summed to yield the beamformed data *y* (*x, z*). Each point (*x, z*) in the beamformed data was weighted using the CF in order to reduce sidelobe levels. The CF can be physically interpreted as the ratio of the coherent energy to the incoherent energy across the receive subaperture. The CF will suppress signals that are incoherent and have low directionality across the array. The CF is defined as

(4) $$CF\left(x_{c},z\right)=\frac{{\left|\sum_{i=1}^{N}s\left(x_{i},z\right)\right|}^{2}}{N\sum_{i=1}^{N}{\left|s\left(x_{i},z\right)\right|}^{2}}$$

where *s*(*x, z*) is the time delayed RF data for element *x_c_* and *N* is the size of the receive subaperture. The final beamformer output was

(5) $$y_{CF}\left(x,z\right)=CF\left(x,z\right)y\left(x,z\right).$$

To find the intensity field *I* (*x, z*), the squared magnitude of the beamformed data *y_CF_*(*x, z*) was summed along a segment of each scan line of length equal to the duration of the visualization pulse using a sliding axial integral at each point (*x, z*). This calculation approximates a pulse intensity integral, which allows one to calculate the intensity of an acoustic waveform given the pressure. The intensity field reconstruction of the FUS beam can be expressed as

(6) $$I\left(x,z\right)=\sum_{i=0}^{L-1}\Delta z{\left|y_{CF}\left(x,z+i\right)\right|}^{2}$$

where *L* is the length of the visualization pulse in samples and Δ*z* is the axial sampling period. Before being displayed, the FUS intensity field was compressed to a logarithmic scale and normalized to the maximum.

### C. Normalization by Echogenicity

The intensity field *I* (*x, z*) of the FUS beam contains information on the position of the beam and the scattering properties of the medium. Strong scatterers will dominate the intensity field reconstruction, pushing the rest of the beam visualization out of the dynamic range used for display. Unlike traditional B-mode imaging, the scattering properties of the medium are not of interest when visualizing the FUS beam, so it is beneficial to equalize *I* (*x, z*) by amplifying regions that correspond to weak scattering. This can be done using the coaligned B-mode image captured immediately after the focused visualization pulse, a process that we refer to as normalizing by echogenicity.

First, a copy of the coaligned B-mode image was smoothed by convolving it with a 5.5 wavelengths × 7 wavelengths (axial × lateral) moving average kernel. This reduced speckle in the B-mode image so that only large-scale features were used to modify the FUS beam visualizations. The magnitude of the signal envelope in the smoothed B-mode image *B*(*x, z*) was used to weight the intensity field *I* (*x, z*). A normalization factor *λ*(*x, z*) was calculated using

(7) $$\lambda \left(x,z\right)=\frac{B_{\max}}{B\left(x,z\right)}$$

where *B_max_* is the maximum value of the signal envelope in the smoothed B-mode image *B*(*x, z*).

The normalization factor can be applied to the beamformed data *y_CF_*(*x, z*) before the pulse intensity integral in (6) is calculated, or it can be equivalently applied to the intensity field *I* (*x, z*) as follows:

(8) $$I_{norm}\left(x,z\right)=I\left(x,z\right)\times L\times {\left|\lambda \left(x,z\right)\right|}^{2}$$

where *L* is the length of the visualization pulse in samples. The factor of *L* accounts for the *L* samples in the pulse intensity integral, and *λ*(*x, z*) is squared because the beamformed data *y*_CF_(*x, z*) are squared to find the intensity.

The scaling factor *λ*(*x, z*) will be close to unity for bright areas in the B-mode image and will be relatively large in dimmer areas. Thus, the FUS beam visualization will be amplified in regions of little scattering, resulting in a more uniform display of the FUS beam. The scaling factor was not applied to points where *B*(*x, z*) was less than −60 dB relative to the maximum because it was assumed that signal from these points was a result of noise and not backscatter.

### D. Experimental Configuration

A 128-element linear array (Ultrasonix L9-4/38; Richmond, BC, Canada) was driven by a Verasonics Vantage 128 Ultrasound System (Kirkland, WA, USA) and used as both an FUS source and as an imaging probe. The array had a center frequency of 5 MHz. When driven as an FUS source, the probe can achieve free field peak negative pressure values up to 2.4 MPa as measured using a needle hydrophone. This is above the pressure values required for many mechanical-based FUS therapies that use low-power sonications with microbubbles. Data were collected from a tissue-mimicking phantom (Supertech Model ATS 539, Elkhart, IN, USA), a wire target in degassed water, and a rat tumor *in vivo.*

An FUS therapy was not carried out, but a mock therapeutic excitation was transmitted to simulate an FUS therapy. The excitation sequence started with a focused 25-cycle tone burst of 5 MHz to simulate a therapeutic tone burst. Next, a focused two-cycle visualization tone burst of 5 MHz was transmitted using the same focusing delays as the mock therapeutic excitation. For all experiments except the one targeting the hypoechoic (−15 dB) contrast target, the visualization pulse and the simulated therapeutic tone burst had a mechanical index of 0.54 (peak negative pressure of 1.21 MPa) as measured using a needle hydrophone in a tank of degassed water. For the FUS beam targeted to the hypoechoic (−15 dB) contrast target, the visualization pulse and therapeutic tone burst had a mechanical index of 0.86 (peak negative pressure of 1.92 MPa) as measured in degassed water. A higher mechanical index was needed for suitable beam visualization in the hypoechoic region because the less backscattered signal was received from the target region. For actual FUS therapy, the therapeutic excitation would be at a higher mechanical index than the visualization excitation. Finally, a series of seven steered plane waves (−18° to 18°) at 5 MHz was transmitted for the formation of a compressed B-mode image. Each of the transmit events used all 128 elements of the array.

The DAS-CF beamformer and pulse intensity integration were implemented using the parallel programming platform CUDA on a GPU (NVIDIA Quadro P2000, Santa Clara, CA, USA). The normalization by echogenicity was carried out on the GPU using a “gpuArray” in MATLAB (MathWorks, Natwick, MA, USA). The built-in Verasonics beamformer was used to create the B-mode images by coherently compounding the steered plane-wave images [41].

### E. Performance Metrics

The FUS intensity field of the L9-4 array was measured in degassed water by sweeping a needle hydrophone through the field using a positioning system (Daedal, Harrison City, PA, USA) with a step size of 200 *μ*m The −3-dB transmit beamwidth and depth of field of the hydrophone-determined FUS intensity field were measured. The −6-dB transmit–receive beamwidth and depth of field were measured for the FUS intensity field determined using ultrasonic backscatter from a homogeneous region of a tissue-mimicking phantom (Model ATS 539 multipurpose phantom). These values were compared with the theoretical −3-dB transmit beamwidth and depth of field assuming a continuous-wave excitation, which is approximated by

(9) $$R_{-3dB}=1.028\lambda f_{\#}$$

(10) $$DOF_{-3dB}=7.08\lambda {\left(f_{\#}\right)}^{2}$$

where *λ* is the wavelength of the excitation and *f_#_* is the f-number of the FUS source [42].

Because the beam visualization method relies on ultrasonic backscatter, it is important to understand how the scattering properties of the medium affect the beam reconstruction. To quantify the fidelity of the reconstruction from one location to the next, mean square error (MSE) values between baseline FUS intensity fields measured from the homogeneous region of a phantom and fields measured from nonhomogeneous regions were calculated. The baselines were defined as FUS intensity fields measured in homogeneous regions of a phantom using the same sequence parameters as the corresponding data measured in nonhomogeneous regions. The intensity fields were converted to decibel scale, normalized to maximum, and clipped to [−60 dB, 0 dB] prior to calculating the MSE so that the data better represented the beam visualization that is displayed. To quantify the performance of normalizing by echogenicity, the MSE values before and after normalization for each nonhomogeneous region were compared. MSE values calculated using five different homogeneous regions were averaged, and all reconstructions were filtered with a 5.5 wavelengths × 5 wavelengths median filter to better control for differences caused by speckle variation. The MSE values between the five sets of baseline data were also calculated to determine the baseline error caused only by speckle variation.

### F. Animal Experiment

The protocol was approved by the Institutional Animal Care and Use Committee (IACUC) at the University of Illinois at Urbana–Champaign. MAT tumor cells (100 *μ*L containing 5 × 10^2^−1 × 10^5^cells) were injected into the mammary fat pad of a rat. Although a tumor was not necessarily required for this work, the animal was also on another protocol that required tumor injections. Once the tumor reached about 1.5 cm in diameter, the animal was scanned using the FUS beam visualization technique. While scanning, the rat was anesthetized with isoflurane.

## III. Results

**Fig. 2.**
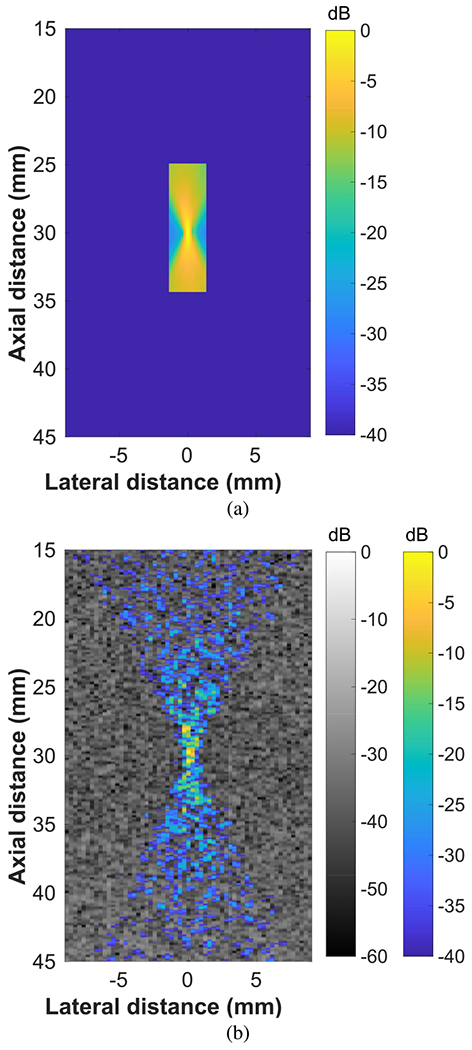
Intensity field of FUS beam targeted to 30 mm using all 128 elements of L9-4 array. (a) Hydrophone determined intensity field. (b) Intensity field reconstructed using ultrasonic backscatter overlaid onto coaligned B-mode image in homogeneous phantom.

The FUS intensity field of the L9-4 array as measured using a needle-hydrophone [see Fig. 2(a)] and ultrasonic backscatter with our method in a homogeneous tissue-mimicking phantom [see Fig. 2(b)] were compared. Based on the hydrophone measurements, the estimated −3-dB transmit (−6-dB transmit–receive) beamwidth and depth of field were 0.35 and 2.05 mm, respectively, and the theoretical values were 0.23 and 1.20 mm. It was difficult to accurately estimate these parameters for the FUS intensity field reconstruction in a homogeneous phantom using the results of our method because speckle-noise degraded the reconstruction, but the estimated values for the −6-dB transmit–receive beamwidth and depth of field were 0.30 and 2.61 mm. We expected differences in the estimated intensity fields of the two methods because the point spread function of the reconstruction based on our method involved properties of the array on receive and transmit, while the hydrophone measurements were transmit only. While our method was not well-suited for quantitative measurements of FUS beam properties, it did provide real-time qualitative information on the beam position in the context of anatomical information provided by the registered B-mode image.

**Fig. 3.**
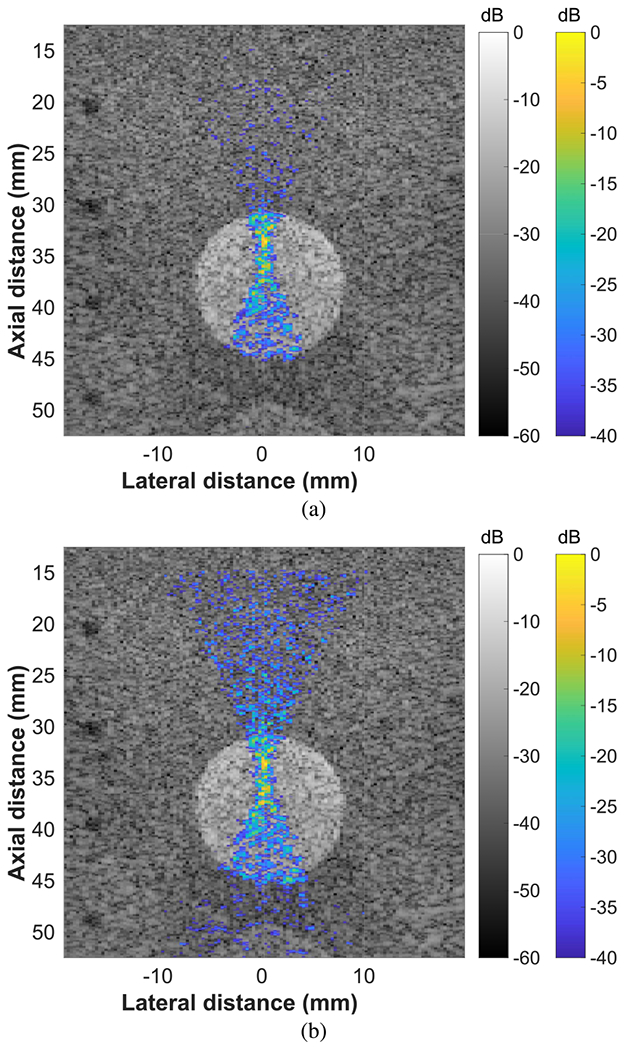
Beam visualizations of an FUS beam targeting a hyperechoic (+15 dB) contrast target in a tissue-mimicking phantom. The FUS beam was targeted to 35 mm using all 128 elements. (a) Before normalizing by echogenicity. (b) After normalizing by echogenicity.

To investigate the effects of variations in the scattering properties of the medium, FUS beam reconstructions were captured in nonhomogeneous regions of a tissue-mimicking phantom. In Fig. 3, beam visualizations are shown for an FUS beam targeted to a hyperechoic (+15 dB) contrast target. Before normalization, the backscatter from the strongly scattering contrast target dominated the dynamic range used for the display of the FUS beam [see Fig. 3(a)]. After normalization, regions outside of the contrast target were amplified such that the entire FUS beam could be visualized [see Fig. 3(b)].

**Fig. 4.**
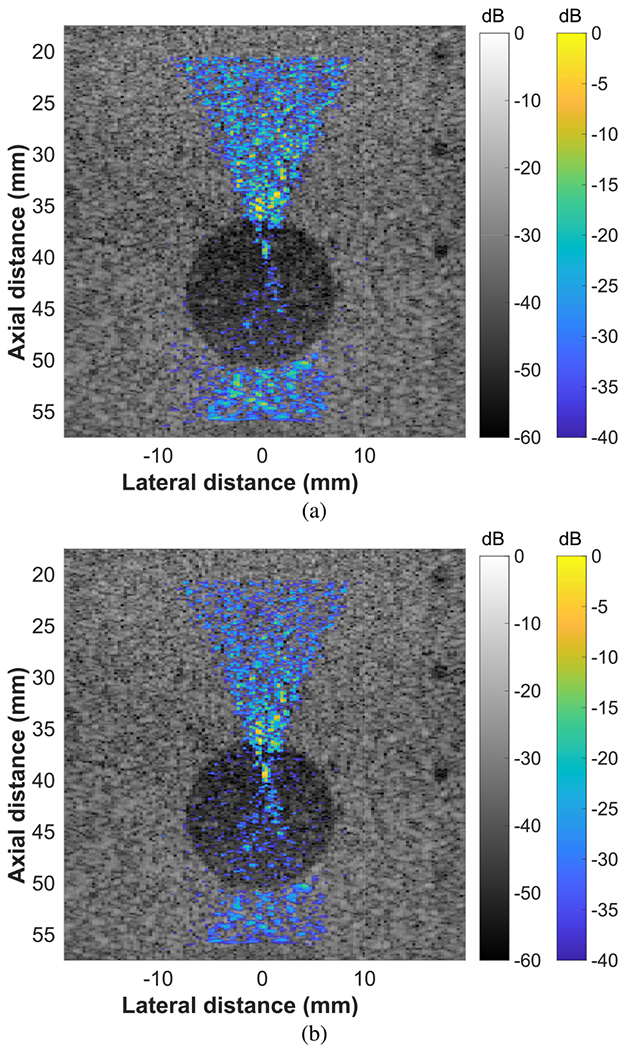
Beam visualizations of an FUS beam targeting a hypoechoic (−15 dB) contrast target in a tissue-mimicking phantom. The FUS beam was targeted to 40 mm using all 128 elements. (a) Before normalizing by echogenicity. (b) After normalizing by echogenicity.

In Fig. 4, the effects of normalization are shown for a reconstruction of an FUS beam targeted to a hypoechoic (−15 dB) contrast target in a tissue-mimicking phantom. Without normalization, there was inadequate visualization of the FUS beam inside the contrast target because little backscatter was received from the hypoechoic region [see Fig. 4(a)]. Normalization was used to amplify the beam reconstruction within the contrast target for better visualization of the FUS beam [see Fig. 4(b)]. This region was deep in the phantom and was hypoechoic. Therefore, a higher pressure excitation was used for the focused beam visualization pulse than that of the other images presented.

**Fig. 5.**
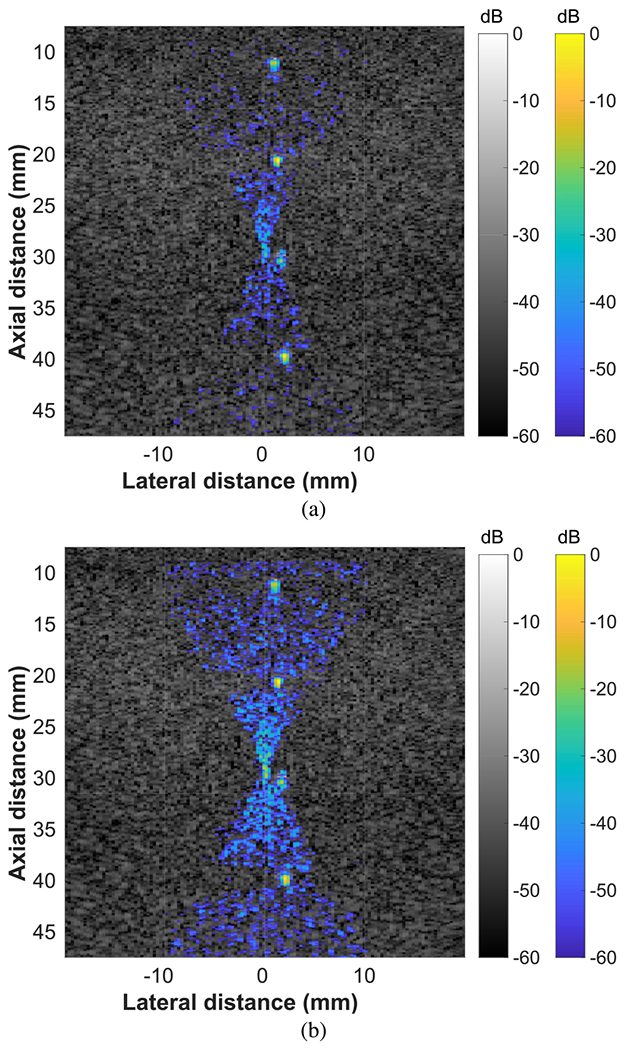
Beam visualizations of an FUS beam targeting a line of point targets in a tissue-mimicking phantom. The FUS beam was targeted to 29 mm using all 128 elements. (a) Before normalizing by echogenicity. (b) After normalizing by echogenicity.

Reconstructions of an FUS beam targeted to a line of point targets are shown in Fig. 5. Before normalization, the strongly scattering point targets dominated the dynamic range of the FUS beam reconstruction [see Fig. 5(a)]. Normalization facilitated easier visualization of the full FUS beam by amplifying regions of the reconstruction outside of the point targets [see Fig. 5(b)]. The regions in the FUS beam reconstructions laterally adjacent to the point targets were darkened. This is an artifact introduced by CF weighting. These regions were attenuated by the CF because the array data corresponding to the regions were dominated by sidelobe signal from the point targets. This dark region effect around hyperechoic targets is a well-known artifact caused by CF weighting [43], [44].

**Fig. 6.**
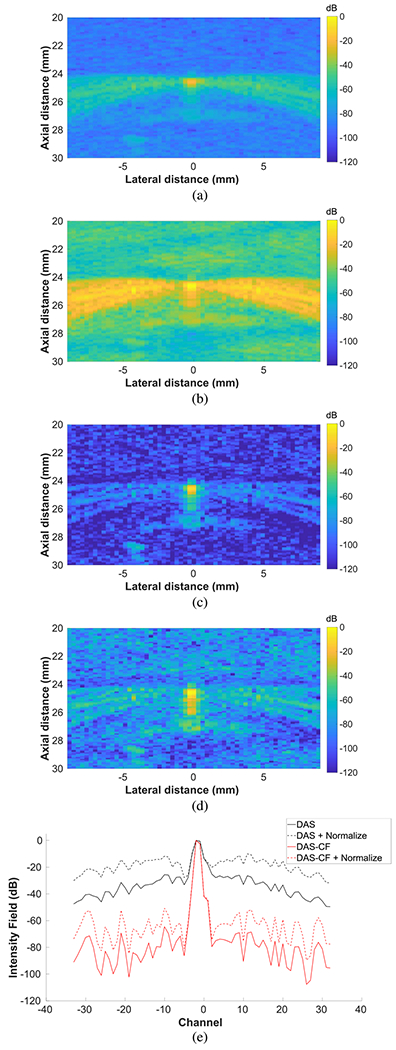
FUS intensity fields measured using a wire target in degassed water. The FUS beam was targeted to 27 mm using all 128 elements. The lower threshold of −60 dB for applying normalization was turned off for these images allowing the normalization factor to be applied to the entire FUS intensity field. (a) DAS beamforming without normalization. (b) DAS beamforming with normalization. (c) DAS-CF beamforming without normalization. (d) DAS-CF beamforming with normalization. (e) Comparison of lateral slice of intensity field at depth of wire target for four images in (a)–(d).

While normalizing by echogenicity was necessary to achieve satisfactory beam reconstructions in nonhomogeneous regions, it resulted in a decrease in the lateral resolution that would degrade image quality when suitable sidelobe reduction steps were not taken. The effects of normalization on sidelobe levels in the FUS beam reconstructions are shown in Fig. 6 along with a comparison of DAS and DAS-CF beamforming. Before normalization, the FUS intensity field from a wire target in degassed water had substantially higher sidelobe levels when DAS beamforming was used [see Fig. 6(a)] compared with DAS-CF beamforming [see Fig. 6(c)]. After normalization, DAS beamforming [see Fig. 6(b)] still performed worse than DAS-CF beamforming [see Fig. 6(d)], and sidelobe levels significantly increased in both images due to normalization. The normalization factor was calculated from the coaligned B-mode image, which resolved the wire target with low sidelobe levels because plane-wave coherent compounding was used. Therefore, the regions corresponding to sidelobes in the FUS beam reconstruction were relatively dark in the B-mode image and were amplified by the normalization factor compared with the main lobe signal that overlapped with the wire target. A comparison of the lateral profile of the FUS intensity field at the depth of the wire target for the four cases reveals that DAS-CF was necessary to reduce sidelobes to an acceptable level because sidelobe levels increased by about 15 dB when normalization was used [see Fig. 6(e)]. The lower threshold of −60 dB for applying normalization was turned off for these results in order to characterize the worst case scenario of sidelobe increase due to normalization.

**Fig. 7.**
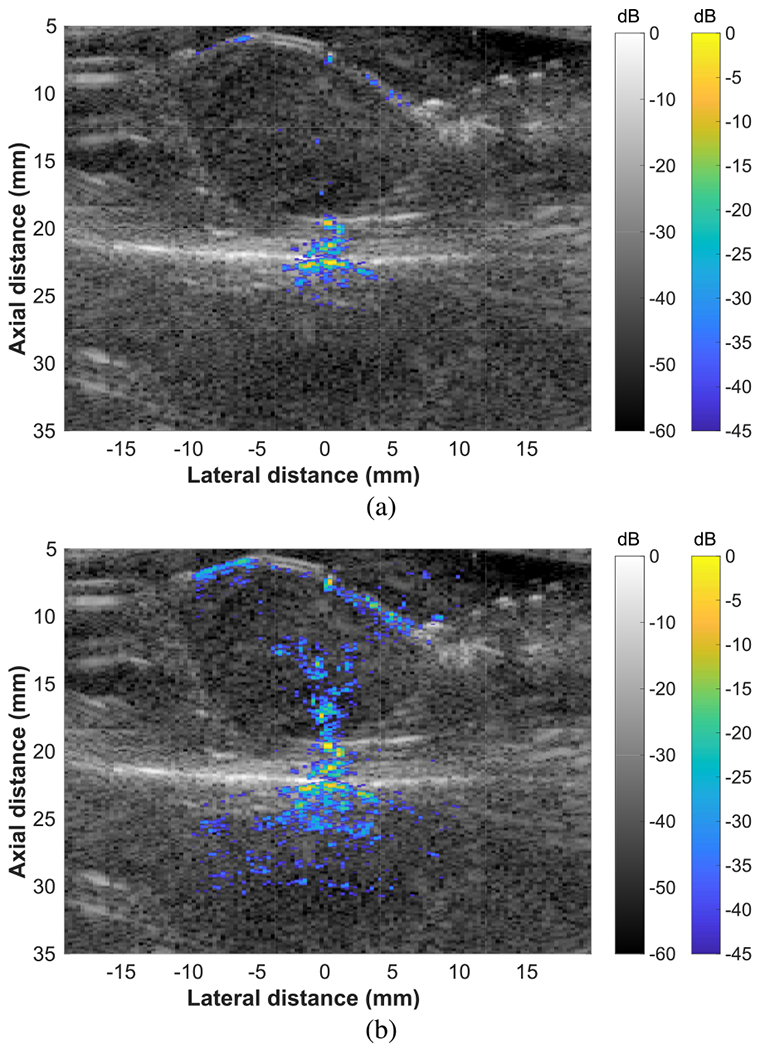
Beam visualizations of an FUS beam targeting a rat tumor *in vivo.* The FUS beam was targeted to 21 mm using all 128 elements. (a) Before normalizing by echogenicity. (b) After normalizing by echogenicity.

FUS beam visualizations were demonstrated in a rat tumor *in vivo* (see Fig. 7). After normalization, the FUS beam can be clearly observed within the context of the surrounding tissue (see Fig. 7(b)). The tumor was centered at approximately 12 mm axially and −3 mm laterally in the B-mode image, and the FUS beam was targeted to the bottom edge of the tumor.

Table 1 lists the MSE values for beam reconstructions in nonhomogeneous regions of a phantom compared with baseline reconstructions in different homogeneous regions. The reported values are an average of MSE values calculated using five different baseline reconstructions. The baseline MSE caused by speckle variation (i.e., average error between different baseline reconstructions) was 14.44 after normalization. For the nonhomogeneous regions, normalizing by echogenicity reduced the MSE by 21.61, on average, indicating that it helped correct for changes in the beam visualization caused by variations in the scattering properties of the medium.

Videos depicting real-time visualization of an FUS beam in a tissue-mimicking phantom can be found in the Supplementary Material. One video shows an FUS beam being manually moved throughout a phantom without normalization (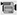), and the other video shows the same scenario but with normalization (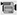). A frame rate of 25–30 frames/s was achieved. Near the end of these recordings, the focal depth and lateral focus of the FUS beam were changed, and the beam visualization was updated in real time. It is worth noting that, during therapy, the amplitude of the visualization excitation should be increased as the focal depth increases, but, for these videos, the amplitude was fixed for all focal depths.

## IV. Discussion

We have presented a technique for the real-time visualization of an FUS beam using ultrasonic backscatter. This method allows one to continuously monitor the extent and position of an FUS beam relative to the surrounding medium. This technique could aid in monitoring FUS therapies because it is important to have real-time information on the position of the FUS beam during therapy so that the focus does not drift outside of the target region. While mechanical-based therapies were the target application of the proposed method, thermal-based therapies could also benefit from real-time beam visualization. MRI thermometry and FUS beam visualization using ultrasonic backscatter could both be used during a thermal-based therapy for additional redundancy and verification of either method.

An FUS beam reconstruction generated using our method in a homogeneous phantom was compared with the intensity field measured using a needle-hydrophone (see Fig. 2). The position and size of the beam in the FUS beam visualization aligned well with those measured using a hydrophone, but the −6-dB transmit–receive beamwidth and depth of field were difficult to estimate in the beam visualization due to the presence of speckle. Frequency compounding was tested as a method to reduce speckle, but it resulted in an overall worse beam reconstruction due to a loss of axial resolution. While our method cannot accurately measure the beamwidth or depth of field of an FUS beam, it does provide real-time information on the position of the beam, which is sufficient for the qualitative monitoring of mechanical-based FUS therapies. Spatial compounding could be used as in [35] to reduce speckle and obtain better estimates of the beam properties, but this was not explored because real-time qualitative beam visualization was the main goal of this work as opposed to quantitative beam measurements.

FUS beam reconstructions were achieved in nonhomogeneous regions of a tissue-mimicking phantom by normalizing the reconstructions based on the echogenicity of the coaligned B-mode images (see Figs. 3–5). Because the FUS beam visualizations were created using ultrasonic backscatter, variations in the scattering properties of the medium affected the estimated intensity field of the FUS beam. The coaligned B-mode images contained information on the scattering properties of the media, allowing effective equalization of the FUS beam reconstructions for better visualization of the full beam field. While a single probe was used for this work, the same normalization process could be used with a separate imaging array and FUS source if the two transducers are registered correctly. It is unlikely that tissue motion will affect the alignment between the B-mode image and the FUS beam reconstruction because the focused visualization pulse and B-mode imaging sequence are executed in quick succession at real-time speeds.

While normalization was necessary to visualize FUS beams in nonhomogeneous regions, it had the unintended consequence of increasing sidelobe levels and degrading lateral resolution (see Fig. 6). Normalizing by echogenicity effectively reduced the tolerance for allowable sidelobes because a normally acceptable level of sidelobes was amplified by up to 15 dB after the normalization was applied. Thus, DAS-CF beamforming was used to suppress sidelobes in the FUS beam reconstructions. CF weighting is one of many adaptive beamforming techniques for the suppression of sidelobes, and there have been some improvements on the CF, such as the generalized CF [45] and the Wiener postfilter [44]. The CF was used here because it is relatively computationally inexpensive, which was important in order to achieve real-time visualization. Because the CF reduces signals that suffer from focusing errors, one drawback is that it may reduce poorly focused signals that we want to visualize. For example, reflections of the FUS beam from tissue interfaces will be subject to focusing errors and suppressed by CF weighting during beamforming because they occur later in time than from the depth they actually originate.

Normalization was shown to, on average, decrease the MSE between baseline reconstructions and reconstructions in nonhomogeneous regions of a phantom (see Table I). This suggests that normalization helped reduce the effects that scattering properties of the medium had on the beam visualizations. However, there are a few limitations with using the MSE to quantitatively compare beam visualizations from different regions of a phantom. First, speckle variance will significantly contribute to the MSE. Even after passing the reconstructions through a median filter to reduce speckle, the baseline MSE caused by speckle variance was found to be 14.44. Second, a beam reconstruction in a homogeneous region is not a true baseline for reconstruction in a nonhomogeneous region because the scattering properties of the medium will affect the actual FUS intensity field. For example, reflective interfaces could result in reflections of the FUS beam and local increases in the intensity that does not exist in reconstructions from homogeneous regions.

A single diagnostic imaging array was used as both an FUS source and an imaging probe for FUS beam visualization. Most ultrasound-based therapy monitoring techniques use multiple transducers although, recently, a mechanical-based FUS therapy was monitored using PCI with a single diagnostic imaging array [34]. Diagnostic imaging arrays are attractive options for FUS therapy transducers because they allow for easy beam steering and image guidance during therapy. These arrays are not engineered for high-power excitations, such as those needed for thermal-based treatments or histotripsy, but they can be suitable for many mechanical-based FUS therapies where low-power sonications are used in conjunction with microbubbles. However, the FUS beam visualization technique presented here does not require the use of a diagnostic imaging array as the FUS source. An FUS source can be used with a secondary imaging array to passively receive backscatter as long as a few requirements are met. Ideally, the two transducers should have an overlapping bandwidth, and the higher the bandwidth of the FUS source, the better, because the axial resolution of the beam reconstruction depends on the length of the focused visualization pulse that the FUS source transmits. The two transducers must be properly aligned with the field of view of the imaging array containing the focal region of the FUS source. Many therapeutic systems use a low-frequency, narrow-bandwidth FUS source with a higher frequency imaging array. For these systems, it is possible that the proposed method could be used with the imaging array passively receiving harmonics of the focused visualization pulse, similar to tissue harmonic imaging [46]. However, this will likely require increasing the mechanical index of the focused visualization pulse, and it is not clear how well the visualization of the harmonic beam will be representative of the actual FUS therapy beam at the fundamental. This technique could also be implemented using a dual-mode FUS source and imaging array, which have successfully been used for real-time B-mode imaging and ultrasound thermometry during therapy [47], [48].

There are a few limitations of the proposed method, specifically relating to the use of a low-power, short-duration visualization pulse to monitor the therapy beam. It is assumed that the focused visualization beam and the focused therapy beam have similar acoustic fields, allowing one to monitor the therapy beam by tracking the position of the visualization beam. However, for therapies where high-power excitations are used, such as thermal ablation and histotripsy, the therapy beam will propagate in a highly nonlinear manner. In these cases, errors will be introduced by assuming that the linear acoustic field of the visualization beam is representative of the nonlinear acoustic field of the therapy beam [49]. Furthermore, the visualization pulse will have different frequency content than the therapy pulse, which is typical of longer duration. The visualization beam is expected to have a broader main lobe than the therapy beam because the short-duration visualization pulse uses a wider bandwidth.

The proposed method is favorable compared with therapy monitoring with only conventional B-mode imaging because it can be used to monitor and confirm the position of the FUS beam before therapeutic effects are induced. B-mode imaging guidance has been shown to be effective for histotripsy by observing the formation of a hyperechoic region during treatment [23]. However, this hyperechoic region signifies that the therapeutic effects (tissue fractionation) are in the process of being produced. This means that B-mode imaging can indicate a misaligned FUS beam but only after damage to the targeted region has occurred. On the other hand, FUS beam visualization using ultrasonic backscatter could be used to confirm that the beam is correctly targeted before any tissue damage occurs.

Although similar to PCI, the beam visualization method presented here is distinct in a number of ways. First, PCI resolves signals from nonlinear microbubble activity caused by the intersection of the therapeutic FUS beam with microbubbles. On the other hand, our method uses backscatter caused by the intersection of the visualization FUS beam with tissue at the fundamental frequency of the transducer. PCI can rely on either relative time-of-flight information [25] or absolute time-of-flight information [30], [33]. When relative time-of-flight is used, the axial resolution of the image is typically poor and relies on the diffraction pattern of the receiving array [25]. When absolute time-of-flight is used, the imaging capabilities of PCI rely on the type of mechanical-based FUS therapy being carried out. If a relatively long excitation is used for FUS therapy, then the axial resolution of PCI suffers. Using our method, the imaging resolution is decoupled from the therapy parameters because separate therapy and beam visualization excitations are used. Second, the microbubble activity that PCI resolves is correlated with the position of the FUS beam and therapeutic bioeffects, which are important to monitor during therapy. Our method can be used only for monitoring of the FUS beam’s position, so passive cavitation detection may still be necessary in some cases if quantitative monitoring of bioeffects is desired. Third, PCI can be used for monitoring when therapeutic effects are being induced (i.e., during therapy), while our method can be used at any time because no microbubble activity is required for visualization of the FUS beam. For therapies where PCI is required for quantitative monitoring of bioeffects, our technique could still be useful for pretherapy alignment of the FUS source and for imaging of the FUS beam at the fundamental frequency during therapy.

## V. Conclusion

We have presented a technique for the real-time visualization of an FUS beam. Ultrasonic backscatter was used to reconstruct the intensity field of an FUS beam, which was overlaid onto a coaligned B-mode image to provide information on the location of the FUS beam in the context of the surrounding tissue. A single diagnostic imaging array was used as an FUS source and for monitoring of the FUS beam. No FUS therapies were carried out, but the excitation sequence contained a mock therapy tone burst to simulate a mechanical-based FUS therapy. FUS beam visualizations were achieved in nonhomogeneous media by applying a normalization factor based on the brightness of the coaligned B-mode image that equalized the FUS beam across regions of varying scattering properties. FUS beam reconstructions were presented in nonhomogeneous regions of a tissue-mimicking phantom and in a rat tumor *in vivo.* FUS beam visualizations reconstructed using ultrasonic backscatter could be used for the planning and real-time monitoring of FUS therapies.

## Supplementary Material

supp 1

supp 2

## Figures and Tables

**TABLE I:** MSE Values for FUS Beam Reconstructions From Different Regions of Phantom Compared With Baseline Reconstructions

Reconstruction	Original	Normalized
Baseline	14.32	14.44
Hyperechoic contrast target (+6 dB)	18.92	14.23
Hyperechoic contrast target (+15 dB)	82.16	21.70
Hypoechoic contrast target (−6 dB)	16.68	16.71
Hypoechoic contrast target (−15 dB)	40.05	34.26
Point targets	82.85	45.72
